# Exceptional points and asymmetric mode conversion in quasi-guided dual-mode optical waveguides

**DOI:** 10.1038/srep19837

**Published:** 2016-04-22

**Authors:** S. N. Ghosh, Y. D. Chong

**Affiliations:** 1 School of Physical and Mathematical Sciences, Nanyang Technological University, 21 Nanyang Link, Singapore 637371; 2 Institute of Radio Physics and Electronics, University of Calcutta, Kolkata-700009, India

## Abstract

Non-Hermitian systems host unconventional physical effects that be used to design new optical devices. We study a non-Hermitian system consisting of 1D planar optical waveguides with suitable amount of simultaneous gain and loss. The parameter space contains an exceptional point, which can be accessed by varying the transverse gain and loss profile. When light propagates through the waveguide structure, the output mode is independent of the choice of input mode. This “asymmetric mode conversion” phenomenon can be explained by the swapping of mode identities in the vicinity of the exceptional point, together with the failure of adiabatic evolution in non-Hermitian systems.

Over the years, many ideas from quantum mechanics have inspired the design of photonic structures, such as photonic crystals; recently, photonics researchers have also drawn ideas from non-Hermitian quantum mechanics^1^,^2^. One particularly interesting phenomenon occurring in non-Hermitian systems is the *exceptional point* (EP): a point in parameter space where the Hamiltonian becomes defective, and two eigenstates coalesce^3^,^4^. The behavior of a non-Hermitian system in the vicinity of an EP is richer than the “avoided level crossings” of Hermitian systems near eigenvalue degeneracies. By encircling an EP in parameter space, one can transition continuously between different branches of the Hamiltonian’s eigenvalues and eigenvectors: the EP acts as a second-order branch point for eigenvalues, and a fourth-order branch point for the eigenvectors.

Several occurrences of EPs in optics and photonics have recently been explored, using partially pumped laser systems, coupled microcavities, and stadium microcavities^5^,^6^,^7^,^8^,^9^,^10^,^11^. In this context, non-Hermiticity is attained by adding loss and/or gain to the optical medium, and the EP is reached by tuning a pair of real parameters, such as geometrical parameters or the amount of gain or loss^12^. The first experimental study of the effects of encircling an EP was reported by Dembowski *et al*.^8^. The possibilities of EPs for mode conversion have been particularly enticing: by exploiting the presence of an EP for two coupled modes, one can in principle convert any order of mode to its coupled counterpart, of either higher or lower order^9^,^11^. Apart from the possibility of technological applications, photonics is a highly useful platform for studying the fundamental physics of EPs, owing to the precise fabrication control and wide tunability available in photonic devices^13^.

In this paper, we explore using linear dual-mode planar optical waveguides for realizing tunable EPs, which can be exploited to achieve controllable on-chip mode conversion. In the photonics community, waveguide structures with balanced of loss and gain regions have been used to achieve parity-time (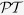) symmetry^1^,^14^,^15^,^16^,^17^; the 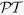-breaking transition is a known example of an EP, but 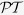 symmetry is not the only way to realize EPs, and in this paper we will not be constrained to 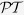 symmetry. We show that a non-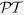-symmetric waveguide can exhibit an EP by tuning the gain level and the gain-to-loss fraction. An encircling of the EP can be realized via a spatial variation in the gain/loss profile. When light passes through the resulting waveguide, it is converted into one specific mode, regardless of the choice of input mode. This “asymmetric mode conversion” results from the rapid variation of eigenstates around the EP and the breakdown of adiabaticity in non-Hermitian systems^10^,^18^,^19^,^20^,^21^. We show also that the effect is robust against small spatial fluctuations in refractive index modulation, so long as the paraxial limit is preserved and the overall spatial variation corresponds to an encircling of the EP. This scheme may have future applications in the design of planar optical waveguides and mode convertors.

## Results and Discussions

### Exceptional Points

An exceptional point is a special type of degeneracy occurring in a non-Hermitian system^3^. It can be accessed by tuning the system through a 2D parameter space (or a single complex parameter); upon reaching the EP, two eigenvectors of the Hamiltonian coalesce, and hence the Hamiltonian becomes defective. A simple model of an EP is given by a 2 × 2 Hamiltonian


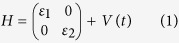


where *ε*_1_ and *ε*_2_ are the eigenvalues of an unperturbed Hermitian Hamiltonian, and


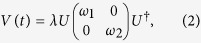



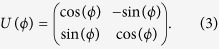


If we let *λ* be complex, the perturbation becomes non-Hermitian, and the eigenvalues are


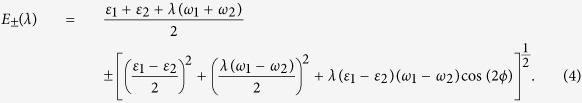


EPs occur at the branch point of *E*_±_(*λ*), arising from the complex square root. They can be accessed by setting the complex variable *λ* to


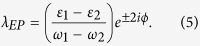


### Optical waveguide with an EP

**Figure 1:**
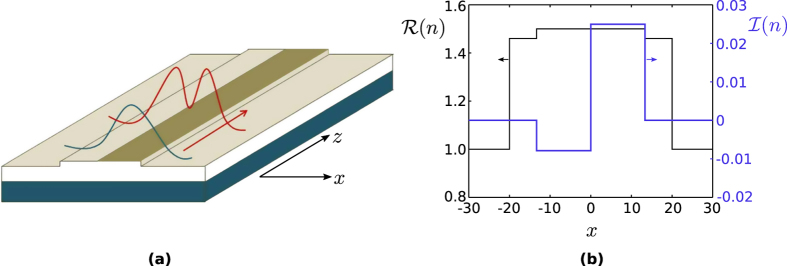
(**a**) Schematic of the waveguide; (**b**) transverse refractive index profile, showing the real (black curve) and imaginary parts (blue curve), using the parameters *γ* = 0.0079 and *τ* = 3.1605.

We now wish to locate an EP in a non-Hermitian photonic structure. Specifically, we consider the planar waveguide shown in Fig. 1(a), with suitably-tailored transverse profile of gain and loss. Let *z* denote the waveguide’s propagation axis, and *x* the transverse direction. For a steady-state mode with frequency *ω*, propagation constant *β*, and transverse mode profile Ψ(*x*),


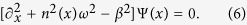


The function *n*(*x*) is the transverse profile of the waveguide’s refractive index, which consists of a “core” region surrounded by a “cladding” region:


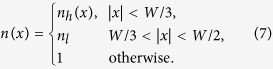


The refractive index of the cladding will be fixed at *n*_*l*_ = 1.46. For the core refractive index, we set Re(*n*_*h*_) = 1.5 and allow for a spatial variation in the imaginary part, to be described below. We also normalize *ω* = 1, and set the total width of the waveguide at *W* = 40 in dimensionless units (i.e., *W* = 20*λ*/*π* where *λ* is the free-space wavelength). Such a structure can be straightforwardly fabricated by thin-film deposition of glass over a thick substrate of silica glass, or standard spin coating. With these parameters, the waveguide supports two guided modes: a fundamental mode (FM) and the first higher-order mode (HOM). A scalar mode analysis is valid so long as the modes are sufficiently well-guided to the core region, since the index difference between the core and cladding regions is small.

Gain and loss are now introduced to the left and right halves of the core region, so that


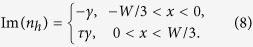


By tuning the two parameters *γ* and *τ*, we can independently adjust the overall non-Hermiticity and the gain/loss ratio. For *τ* = 1, the system is 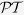-symmetric. For *γ* = 0, the system is Hermitian and the modal propagation constants *β* are real; for *γ* > 0, the modes become amplified or damped “quasi-modes”, with complex *β*.

**Figure 2:**
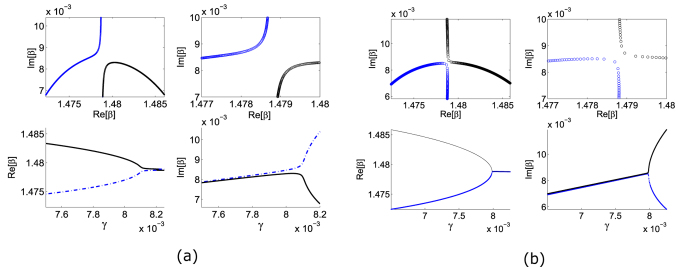
Trajectories of the propagation constants for the two waveguide modes, with increasing gain/loss parameter *γ*. The gain/loss fraction is fixed at *τ* = 3.1 in (**a**), and *τ* = 3.161 in (**b**). In each case, we plot the variation in 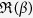 and ℑ(*β*) with *γ*, with block curves representing the fundamental mode and blue curves representing the higher-order mode. The upper panels show a zoomed-in portion of the complex *β* plane, where the *β* trajectories come near each other.

Figure 2 plots the complex values of *β*, for each of the two waveguide modes, as we vary the overall gain/loss parameter *γ*. We observe a richer behavior than the usual “level repulsion” phenomenon occurring in Hermitian systems. For fixed *τ* = 3.1, the propagation constants repel each other in the complex *β* plane, as shown in Fig. 2(a); as we increase *γ*, 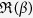 undergoes anti-crossing and ℑ(*β*) undergoes crossing. For a slightly larger value of *τ* = 3.161, the trajectories exchange identities, with 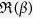 undergoing crossing and ℑ(*β*) undergoing anti-crossing. Because these two behaviors are topologically inequivalent, in between these two values of *τ* there must be a sharp transition where the two modes coalesce at a critical point (*γ*_*EP*_, *τ*_*EP*_). At this point, there is only a single field pattern and propagation pattern representing a guided mode. In this case, we find numerically that the EP occurs at *γ*_*EP*_ = 0.0079, *τ*_*EP*_ = 3.1605.

**Figure 3:**
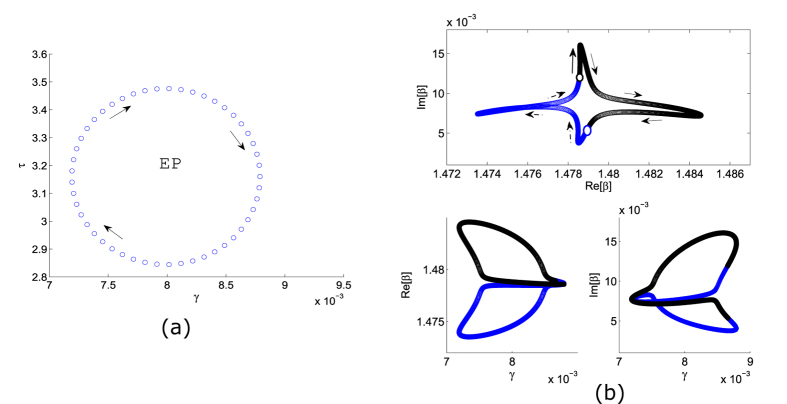
(**a**) Trajectory in the 2D parameter space (*γ, τ*) defined in Eq. (9), centered at and enclosing the EP at *γ*_*EP*_ = 0.0079, *τ*_*EP*_ = 3.1605 with *r* = 0.1. (**b**) Complex trajectories of the propagation constant *β*, for each of the two waveguide modes (shown in blue for the fundmanetal mode, and black for the higher-order mode). The arrows indicate the direction of evolution under a clockwise cycle in the parameter space. The white circles indicate the starting points at Φ = 0.

We now consider the effect of encircling this EP. We choose a closed loop in the 2D parameter space by taking


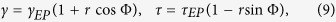


where *r* > 0 is some small radius and Φ is a tunable angle variable. This parameter trajectory is shown in Fig. 3(a). In Fig. 3(b), we show the propagation constants for the two modes under one clockwise loop. As can be seen, this causes the two modes to exchange positions in the complex *β* plane, reflecting the fact that the EP serves as a second-order branch point for the eigenvalues. We must cycle through the parameter loop twice in order for the modes to return to their starting points in the complex *β* plane. By contrast, for a parameter loop that does not enclose an EP, the propagation constants would loop back to themselves after a single cycle.

**Figure 4:**
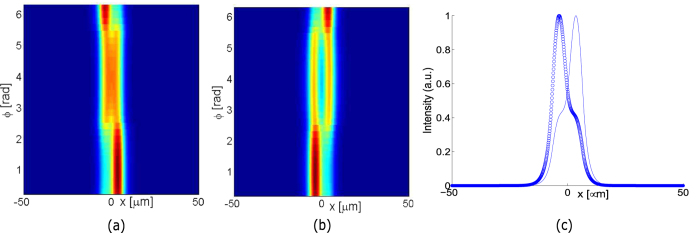
Evolution of (**a**) Ψ_*FM*_ and (**b**)Ψ_*HOM*_ around the EP along the circular loop in the clockwise direction of progression as shown in Fig. 3a; (**c**) Corresponding (**b**) normalized squared mode-fields plotted at the beginning (dotted line) and end of the EP (solid line) encircling for the evolution of Ψ_*FM*_.

During the EP-encircling process, the underlying eigenmodes (i.e. the mode functions) also exchange identities. This is visualized in Fig. 4. In Fig. 4(a,b), we plot the mode intensity profiles |Ψ(*x*)|^2^ for each value of Φ along the loop specified by Eq. (9). (It is important to note that this is *not* a beam-propagation calculation.) From this, we see that the mode intensity profiles are exchanged under one cycle around the EP. In fact, each cycle around the EP the modes also causes one of the modes to undergo a sign flip (e.g. [Ψ_*FM*_, Ψ_*HOM*_] → [Ψ_*HOM*_, −Ψ_*FM*_]), reflecting the fact that the EP is a fourth-order branch point for the eigenmodes.

The exchange of mode identities when encircling an EP is distinctly different from any mode mixing or coupling phenomena occurring in Hermitian systems. At first glance, we might assume that it raises possibility of achieving efficient optical mode *switching*. But as shall later see, this is not achievable due to the breakdown of adiabaticity in non-Hermitian systems^19^. However, we will instead be able to demonstrate asymmetric conversion into a single mode.

### Mapping parameter space evolution to waveguide index variation

In the waveguide geometry, the encircling of an EP in parameter space can be implemented by varying the waveguide’s transverse index profile along the *z* axis. In other words, we must continuously tune the amount of gain and loss in the two halves of the waveguide core, so that for each value of *z* the index profile corresponds to a desired set of (*γ, τ*) lying along the parameter loop. Typically, this mapping requires a slow variation along *z*, so that the modes variation is adiabatic (based on the usual analogy between waveguides in the paraxial approximation and the time-dependent Schrodinger system, where *z* plays the role of the time coordinate).

Previously, we have encircled the EP using the simple circular loop described by Eq. (9), with *r* ≪ 1. For device applications, it is more useful to describe a situation where *γ* = 0 at the inputs and outputs of the waveguide (i.e., no gain or loss). This ensures that the effects of encircling of the EP are applied to the fundamental and higher-order modes of a conventional waveguide, which could then be connected to other optical components. Hence, we replace Eq. (9) with


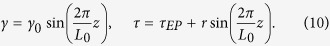


**Figure 5:**
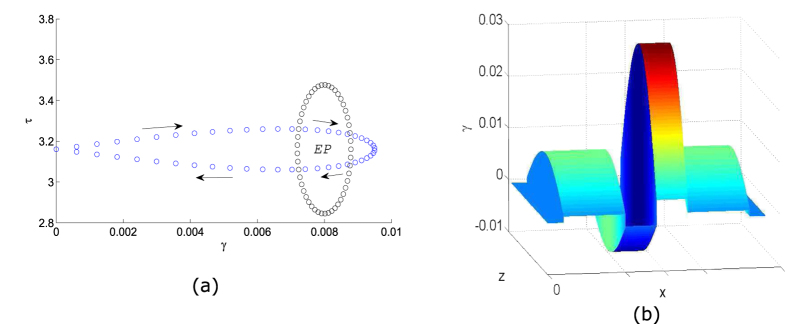
(**a**) Loops in the parameter space described by Eq. (10), with *r* = 0.1 and *γ*_0_ = 0.0095 (blue dots). The circular loop from Fig. 3 is included for comparison (black dots). (**b**) The corresponding variation of ℑ(*n*), the imaginary part of the refractive index profile, with *x* and *z*.

For *γ*_0_ > *γ*_*EP*_ and 0 < *z* < *L*_0_, this describes a parameter space trajectory encircling the EP, as shown in Fig. 5(a). The loop is clockwise for *r* > 0, and anticlockwise for *r* < 0. The corresponding variations in ℑ(*n*) are plotted in Fig. 5(b).

### Asymmetric mode conversion

We now numerically determine the mode evolution dynamics under the EP-encircling scheme described above. If the index variations along *z* are much slower than the wavelength, the (1 + 1)*D* scalar wave equation reduces to the paraxial equation


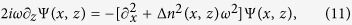


where 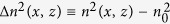, and we use the *z*-dependent parameters specified by Eq. (10). The paraxial equation can be solved numerically with the Split-Step Fourier method^22^.

**Figure 6:**
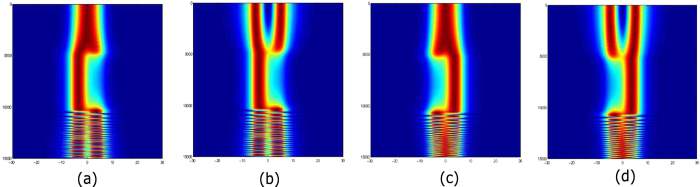
Beam propagation simulation results showing asymmetric mode conversion caused by encircling an EP. (**a,b**) Effects of encircling the EP clockwise, using input (**a**) Ψ_*FM*_ and (**b**) Ψ_*HOM*_. (**c,d**) Effects of encircling the EP anticlockwise, using input (**c**) Ψ_*FM*_ and (**d**) Ψ_*HOM*_. In all cases, the EP encircling is parameterized by Eq. (10) with *γ*_0_ = 0.0095, |*r*| = 0.1, and *L*_0_ = 1.5 × 10^4^. For ease of visualization, the intensities are re-normalized for each *z*, so the overall intensity change is not shown.

The results are shown in Fig. 6. At *z* = 0, the waveguide is initially free of gain or loss, and we input light in the exact fundamental mode (FM) or the higher-order mode (HOM), both of which are bounds with real values of *β*. We set the total device length at 1.5 × 10^4^ in dimensionless units (around 2400 free-space wavelengths). Figure 6(a,b) shows the effects of encircling the EP clockwise (*r* > 0). Regardless of the choice of input mode, the output mode is strongly converted to the HOM at the output *z* = *L*_0_. On the other hand, Fig. 6(c,d) shows the effects of encircling the EP anticlockwise (*r* > 0); in this case, regardless of the choice of input mode, the output is converted to the FM.

The occurrence of asymmetric mode conversion, rather than the mode-switching one might expect from a naive interpretation of the preceding discussion, can be attributed to the breakdown of adiabaticity: a phenomenon that has previously been discussed in detail by Moiseyev and co-workers^18^,^19^. In Hermitian systems, modes can be transported adiabatically so long as the parameter space trajectory is sufficiently slow; however, non-Hermitian systems do not behave this way.

To see how adiabaticity can break down, consider a (possibly non-Hermitian) Hamiltonian 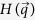, parameterized by a real vector 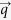. In the case of the simple 2 × 2 Hamiltonian from Eqs. (1)–(3), 12, 13, for instance, 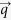 could be the real and imaginary parts of the *λ* parameter; for our waveguide system the same role is played by the gain/loss parameters *γ* and *τ*. We evolve 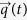 in time, so that the instantaneous eigenstates and eigenenergies at time *t* are 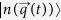 and 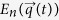. Without loss of generality, the state at time *t* can be written as


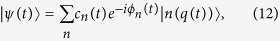


where


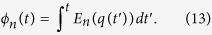


Substituting this into the time-dependent Schrödinger equation gives


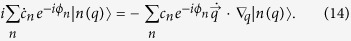


Here, we have suppressed the *t* dependences for notational simplicity. Suppose we prepare the system in an instantaneous eigenstate |*a*〉. If adiabaticity holds, then for sufficiently slow variations in 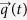 the amplitude *c*_*a*_(*t*) should dominate all the other amplitudes for subsequent times. We can check the self-consistency of this statement by left-multiplying both sides of Eq. (14) by 〈*b*(*q*(*t*))| for some other state *b* ≠ *a*. This gives


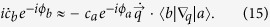


Here, we have assumed that the eigenstates remain approximately power-orthogonal. Hence,


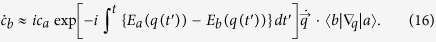


In the usual Hermitian case, the quantity in the exponential is just a phase factor, so we can indeed suppress 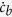 by making 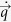 arbitrarily small (i.e., the evolution arbitrarily slow). If, however, the system is non-Hermitian, the quantity in the exponential is *not* generally a phase factor since the eigenenergies need not be real. If this is a growing exponential, then the self-consistency of the above calculation breaks down: as we vary 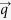 arbitrarily slowly along a loop in parameter space, state *b* will eventually acquire a rapidly growing amplitude.

Returning to the non-Hermitian optical waveguide system, Fig. 6 shows that choice of direction with which we encircle the EP determines whether the output mode is the FM or HOM mode, regardless of the choice of input mode. This is because the choice of direction determines the “connection” between the modes of the intermediate non-Hermitian system and the output modes. As shown in Fig. 3(b), for instance, if clockwise encirclement connects a low-loss intermediate mode to one output mode, anticlockwise encirclement would connect that intermediate mode to the other output mode. Note that in Fig. 6, the intensities are re-normalized for each *z* for ease of visualization, so the overall intensity change is not shown.

The efficiency of the mode conversion depends on the choice of device length *L*_0_. Unlike other mode converters based on adiabatic evolution, the present conversion is not purely adiabatic, so the large-*L*_0_ limit is not unconditionally desirable^23^,^24^. In particular, if the intermediate modes are lossy, it would be desirable to have *L*_0_ shorter than the mode decay length. In Fig. 6, we chose *L*_0_ = 1.5 × 10^4^ in dimensionless units, which corresponds to 3.7 mm for a 1.55μm free-space operating wavelength. For this design, we calculate the conversion efficiency using the overlap integrals between the input and output fields:


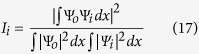


where subscript *i* denotes the choice of input mode (either FM or HOM), and *o* denotes the choice of output mode. In this way, we find conversion efficiencies of 91.72% for conversion of either the FM or HOM into the FM (the two conversion efficiencies differ by less than 0.01%), and 63% for conversion of either the FM or HOM into the HOM.

These conversion efficiencies appear to be robust against perturbations to the path taken in encircling the EP. To test this, we modified the parameter trajectory by adding uncorrelated random fluctuations of up to 10% in both *γ* and *τ*, at each point of the waveguide. Over 100 realizations of the disorder, the conversion efficiency was 91.63% ± 0.63% into the FM, and 62.14% ± 1.44% into the HOM.

In summary, we have studied a robust mechanism for asymmetric mode conversion in non-Hermitian optical waveguides exhibiting exceptional points. The example system consists of dual-mode waveguides on a glass substrate, but a similar scheme could be implemented in other waveguide geometries, including optical fibers. An important limiting factor is the total transmission; if the modes are lossy, as in the example we have considered, the total transmission after a large number of wavelengths may be too weak for a useful device. The time-reverse of the system, in which the modes are amplifying, may thus be more useful for experimental realizations. In that case, the effects of nonlinear gain saturation may introduce novel optical effects, beyond those previously studied in 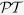 symmetric waveguides.

## Additional Information

**How to cite this article**: Ghosh, S. N. and Chong, Y. D. Exceptional points and asymmetric mode conversion in quasi-guided dual-mode optical waveguides. *Sci. Rep.* **6**, 19837; doi: 10.1038/srep19837 (2016).
